# Impact of Age and Sex on Subclinical Coronary Atherosclerosis in a Healthy Asian Population

**DOI:** 10.1016/j.jacasi.2021.05.002

**Published:** 2021-06-15

**Authors:** Mark Yu Zheng Wong, Jonathan Yap, Weiting Huang, Swee Yaw Tan, Khung Keong Yeo

**Affiliations:** a Department of Cardiology, National Heart Centre Singapore, Singapore; b School of Clinical Medicine, University of Cambridge, Cambridge, United Kingdom; c Duke-NUS Medical School, Singapore

**Keywords:** age, Asian, coronary artery calcification, coronary atherosclerosis, healthy, sex, subclinical disease, BMI, body mass index, CAC, coronary artery calcification, CAD, coronary artery disease, HDL, high-density lipoprotein, LDL, low-density lipoprotein, SBP, systolic blood pressure

## Abstract

**Background:**

The influence of age and sex on clinical atherosclerotic cardiovascular disease is well reported, but literature remains sparse on whether these extend to the disease in its preclinical stage.

**Objectives:**

The purpose of this study was to report the prevalence, risk factors, and impact of age and sex on the burden of subclinical coronary atherosclerosis in a healthy Asian population.

**Methods:**

Healthy subjects age 30 to 69 years, with no history of cardiovascular disease or diabetes were recruited from the general population. Subclinical coronary atherosclerosis was quantified via the coronary artery calcium score (CAC) with CAC of 0 indicating absence of calcified plaque, 1 to 10 minimal plaque, 11 to 100 mild plaque, and >100 moderate to severe plaque.

**Results:**

A total of 663 individuals (mean age 49.4 ± 9.2 years; 44.8% men) were included. The prevalence of any CAC was 29.3%, with 9% having CAC >100. The prevalence was significantly higher in men than women (43.1% vs 18.0%; *P* < 0.001). Multivariable analysis revealed significant associations of increasing age, male sex, higher blood pressure, increased glucose levels, and higher low-density lipoprotein cholesterol levels with the presence of any CAC. Low-density lipoprotein cholesterol was more significantly associated with CAC in women compared with men (*P*_interaction_ = 0.022).

**Conclusions:**

The prevalence of preclinical atherosclerosis increased with age, and was higher in men, with sex-specific differences in associated risk factors. These results will better inform individualized future risk management strategies to prevent the development and progression of coronary artery disease within healthy individuals.

## Central Illustration

**Figure:**
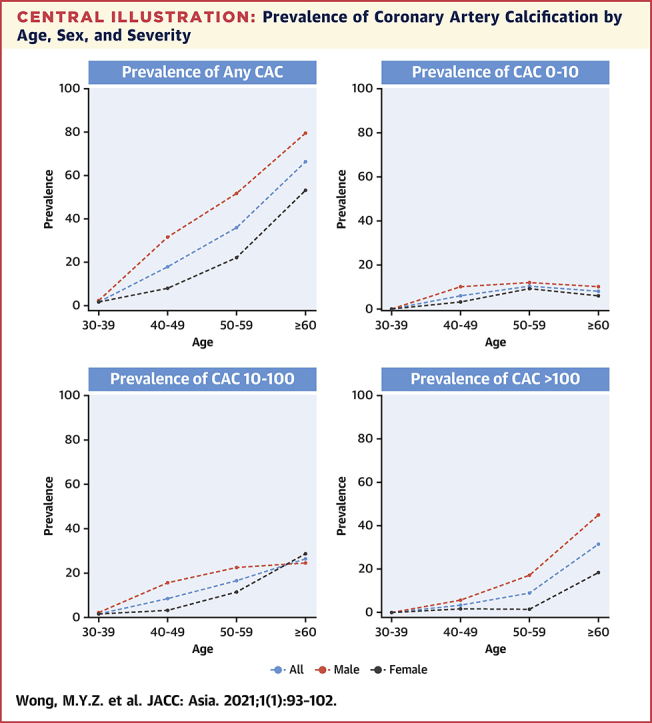

Atherosclerosis is a long, complex process that involves the gradual accumulation of fat, inflammation, scar tissue, and calcium deposits within the walls of arteries (1). Due to the long latent period of atherosclerosis and its initial development during early life (1,2), increasing focus has now been placed on the primordial prevention of atherogenic risk factors, and the identification and possible intervention of this process at its preclinical phase (3).

The influence of age and sex on patterns of coronary artery disease (CAD) is well reported in literature, with epidemiological, clinical, and experimental data providing evidence for sex-specific differences in the development, prevalence, presentation, and progression of CAD (4, 5, 6, 7). In addition, the prevention and treatment outcomes of CAD differ across age and sex (8,9). What is less understood however, is whether—and to what extent—these age and sex differences extend to the extent of coronary atherosclerosis at its preclinical phase. This holds important implications for screening and preventative strategies designed toward the early evaluation and risk assessment of CAD in asymptomatic individuals.

Coronary artery calcium (CAC) is a highly specific and well-established subclinical marker of coronary atherosclerosis, with consistent evidence linking CAC with major cardiovascular outcomes (10, 11, 12, 13, 14, 15). In our present study, we use the CAC score to quantify the extent of preclinical coronary atherosclerosis, and report on the baseline prevalence, associated risk factors, and influence of age and sex on the extent of the preclinical atherosclerotic burden within a healthy Asian population without known cardiovascular disease. We also evaluate the potential utility of the CAC score as a risk enhancing factor in this asymptomatic Asian population.

## Methods

### Study population

SingHEART is a prospective population-based study of healthy Asian adults living in Singapore. The full details have previously been published (16,17). In summary, the SingHEART study aims to evaluate the development of cardiovascular disease among asymptomatic healthy individuals (16). Healthy subjects age 21 to 69 years were recruited from the general population from October 2015 to July 2020 and were enrolled according to approved standardized protocol. Inclusion criteria were as follows:

1. No known history of any prior cardiovascular disease (ischemic heart disease, stroke, or peripheral vascular disease).
2. No known history of any prior cancer, autoimmune/genetic disease, endocrine disease, diabetes mellitus, psychiatric illness, asthma, chronic lung disease, or chronic infective disease.
3. No family medical history of cardiomyopathies

Data on demographics, lifestyle factors, and medical history were collected via standardized questionnaires. Clinical measurements of height, weight, and blood pressure were taken, and fasting lipid and glucose bloods were obtained.

Written informed consent was obtained from all volunteers, and the study was approved by the intuitional ethics review board (SingHealth CIRD ref: 2015/2601).

### CAC scoring

As part of the SingHEART protocol (16), all subjects age ≥30 years underwent a single CAC assessment at baseline. To obtain CAC measurements, noncontrast cardiac computed tomography (CT) scans were performed on a 320 × 0.5-mm detector row CT system (Canon Medical Systems). Prospective ECG triggering was used, and scans covered a single heartbeat with a gantry rotation and x-ray exposure time of 0.35 s and 0.5-mm slice collimation. CAC scores were calculated using the Agatston method (18) via Vitrea Workstation (Vitrea, FX version 3.0 workstation, Vital Images), and classified according to standardized categories, with scores of 0 indicating the absence of calcified plaque, 1 to 10 minimal plaque, 11 to 100 mild plaque, and >100 moderate to severe plaque (14,19).

### Cardiovascular risk classification

The MESA (Multi-Ethnic Study of Atherosclerosis) coronary heart disease (CHD) risk score was used to classify participants by cardiovascular risk. The MESA CHD risk score is an algorithm that incorporates various atherosclerotic risk factors to predict the 10-year risk of CHD (20,21). The score comprises of 2 models, one largely relying on the traditional Framingham risk variables, and another model that additionally incorporates subclinical atherosclerotic risk as measured by the CAC score. The MESA risk scores for all participants were generated both with and without the incorporation of CAC scores. Although stratification thresholds specific to the MESA risks scores have yet to be fully established, a 10-year risk score <7.5% was considered “low” and a score ≥7.5% was considered “elevated,” as per general ACC/AHA cardiovascular risk assessment guidelines (22,23).

### Statistical methods

The prevalence rates of any CAC (CAC >0) and various CAC severity categories (CAC 0 to 10, >10 to 100, >100) were computed for the whole cohort and stratified by age and sex. 95% CIs were calculated assuming that the prevalence counts were binomially distributed.

Differences in traditional cardiovascular risk factors, for participants with and without CAC, were first compared using chi-square statistic for proportions and an independent sample Student's *t*-test/Mann-Whitney *U* test for means or median as appropriate. Analysis of variance was then used to further analyze differences across participants of the varying CAC severities.

Multiple logistic regression models were used to examine the relationships between clinical variables and CAC with OR and 95% CI obtained. Multivariate models were adjusted for a fixed set of traditional cardiovascular risk factors: age, sex, smoking, sedentary behavior (as defined by participant occupation), body mass index (BMI), systolic blood pressure (SBP), glucose, low-density lipoprotein (LDL) cholesterol, and high-density lipoprotein (HDL) cholesterol (24). Total cholesterol and diastolic blood pressure were include in univariate but not multivariate models to avoid collinearity. Further sex-stratified analyses were then performed, with each of the risk factors tested for interaction with sex. As a sensitivity analysis, categorical values for total cholesterol (≥6.2 mmol/L vs <6.2 mmol/L), LDL cholesterol (≥4.1 mmol/L vs <4.1 mmol/L), and triglycerides (≥2.3 mmol/L vs <2.3 mmol/L) using the National Cholesterol Education Program ATP III cutoffs for “high levels” (25) were also included in separate multivariable models.

Last, changes in the MESA risk classification of participants before and after incorporation of CAC scores were evaluated using paired Student’s *t*-tests for continuous variables, and the McNemar’s test for categorical variables. For all analyses, a *P* value <0.05 was considered statistically significant. All statistical analyses were performed using Stata 14.1 (StataCorp).

## Results

A total of 800 participants were recruited, of which 135 (16.8%) were aged <30 years and did not undergo CT evaluation, and a further 2 refused CT evaluation. Of the total included 663 individuals, the mean age was 49. 4± 9.2 years, and 297 (44.8%) were men. All patients had an estimated glomerular filtration rate >55 mL/min/1.73 m^2^.

The overall prevalence of any CAC (CAC >0) was 29.3% (95% CI: 24.9%-32.8%). Of these, 47 (7.1%), 87 (13.1%), 60 (9.0%), and 17 (2.6%) had CAC scores of >0-10, >10-100, >100 and, >400, respectively. The overall prevalence of any CAC was significantly higher across each age group, increasing from 1.9% in those aged 30-39 years to 66.3% in those ≥60 years (*P <* 0.001). In terms of sex differences, the prevalence of any CAC was significantly higher in men than women (43.1% vs 18.0%; P < 0.001) (Central Illustration, Table 1).

**Central Illustration:**
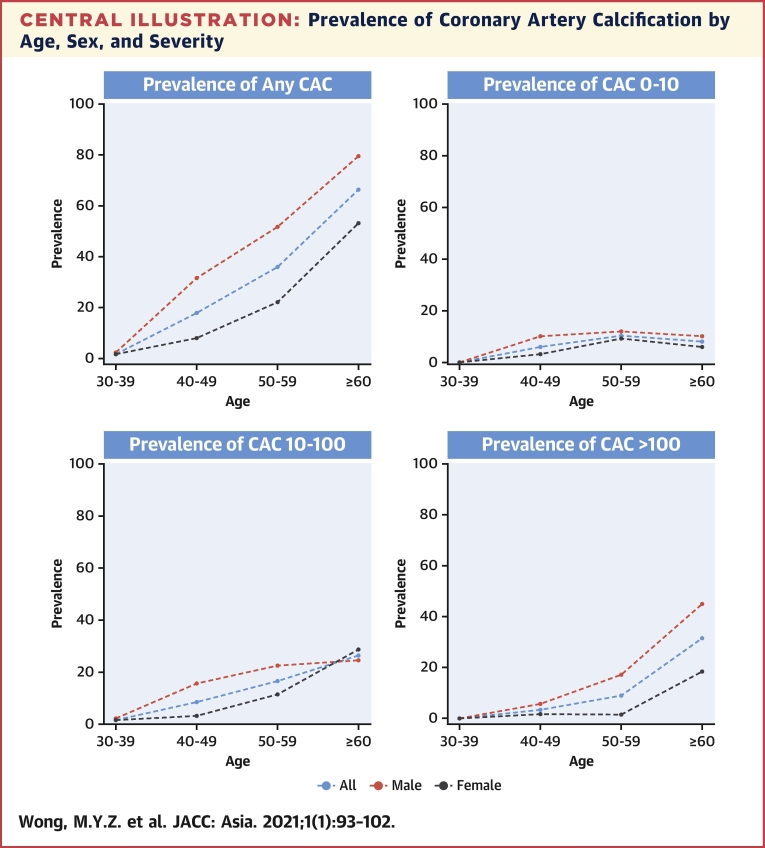
Prevalence of Coronary Artery Calcification by Age, Sex, and Severity The prevalence of varying severities of coronary artery calcification (CAC), stratifying by sex and 10-year age groups.

**Table 1:** Prevalence of CAC by Age, Sex, and Severity

	Overall (N = 663)			Per 10-y Age Strata				*P* Value for Age
	N	Prevalence (95% CI)	*P* Value for Sex	30-39 (n = 105)	40-49 (n = 213)	50-59 (n = 247)	≥60 (n = 98)	
Any CAC								
All	194	29.3 (25.8-32.9)	**<0.001**	2 (1.9)	38 (17.8)	89 (36.0)	65 (66.3)	**<0.001**
Men	128	43.1 (37.4-48.9)		1 (2.33)	28 (31.5)	60 (51.7)	39 (79.6)	**<0.001**
Women	66	18.0 (14.2-22.4)		1 (1.61)	10 (8.06)	29 (22.1)	26 (53.1)	**<0.001**
CAC 0-10								
All	47	7.1 (5.3-9.3)	**0.035**	0 (0.0)	13 (6.1)	26 (10.5)	8 (8.2)	**0.005**
Men	28	9.4 (6.4-13.3)		0 (0.0)	9 (10.11)	14 (12.1)	5 (10.2)	0.138
Women	19	5.2 (3.2-8.0)		0 (0.0)	4 (3.23)	12 (9.16)	3 (6.12)	**0.034**
CAC 10-100								
All	87	13.1 (10.6-15.9)	**0.001**	2 (1.9)	18 (8.5)	41 (16.6)	26 (26.5)	**<0.001**
Men	53	17.8 (13.7-22.7)		1 (2.33)	14 (15.7)	26 (22.4)	12 (24.5)	**0.015**
Women	34	9.3 (6.5-12.7)		1 (1.61)	4 (3.2)	15 (11.5)	14 (28.6)	**<0.001**
CAC >100								
All	60	9.0 (7.0-11.5)	**<0.001**	0 (0.0)	7 (3.29)	22 (8.91)	31 (31.63)	**<0.001**
Men	47	15.8 (11.9-20.5)		0 (0.0)	5 (5.62)	20 (17.2)	22 (44.9)	**<0.001**
Women	13	3.6 (1.9-6.0)		0 (0.0)	2 (1.61)	2 (1.53)	9 (18.4)	**<0.001**

CAC = coronary artery calcification.

Subjects with any CAC were significantly more likely to be older; be men; and have higher SBP, diastolic blood pressure, fasting glucose, total cholesterol, LDL cholesterol and triglyceride levels than those without (all *P <* 0.001). Among those with CAC, participants who were older, were men and who smoked were more likely to have moderate-severe CAC (Table 2).

**Table 2:** Clinical Characteristics of Study Population

	Overall Population	CAC = 0	Any CAC (CAC > 0)	*P* Value
Age (y)	50 (43-56)	47 (41-43)	56 (50-61)	**<0.001**
Male	297 (44.8)	169 (36.0)	128 (66.0)	**<0.001**
Smoking	51 (7.69)	32 (6.82)	19 (9.79)	0.192
Body mass index (kg/m^2^)	23.1 (21.0-25.6)	23.1 (21.0-25.5)	22.9 (20.9-25.7)	0.977
Systolic blood pressure (mm Hg)	126 (115-140)	123 (113-135)	135 (122-147)	**<0.001**
Diastolic blood pressure (mm Hg)	78 (69-87)	76 (68-85)	82.5 (75-90)	**<0.001**
Glucose (mmol/L)	5.2 (5.0-5.5)	5.1 (4.9-5.4)	5.4 (5.1-5.7)	**<0.001**
Total cholesterol (mmol/L)	5.40 (4.76-6.03)	5.29 (4.71-5.94)	5.71 (5.00-6.22)	**<0.001**
LDL cholesterol (mmol/L)	3.36 (2.82-3.9)	3.28 (2.76-3.85)	3.60 (3.09-4.10)	**<0.001**
HDL cholesterol (mmol/L)	1.46 (1.24-1.71)	1.47 (1.26-1.70)	1.43 (1.19-1.74)	0.416
Triglycerides (mmol/L)	1.00 (0.74-1.40)	0.95 (0.71-1.34)	1.13 (0.87-1.58)	**<0.001**
Total cholesterol ≥6.2 mmol/L	125 (18.9)	75 (16.0)	50 (25.8)	**0.003**
LDL cholesterol ≥4.1 mmol/L	122 (18.4)	73 (15.6)	49 (25.3)	**0.003**
Triglycerides ≥2.3 mmol/L	41 (6.18)	25 (5.33)	16 (8.25)	0.156

Values are median (interquartile range) or n (%). Chi-square (for categorical variables) and independent sample Student's *t*-test/Mann-Whitney *U* (for continuous variables) were used to compare differences.

CAC = coronary artery calcification; HDL = high-density lipoprotein; LDL = low-density lipoprotein.

With regard to the predictors of any CAC, after multivariable adjustment, age (OR: 1.13; 95%CI 1.10-1.17), male (OR: 3.45, 95%CI 2.19-5.43), SBP (OR: 1.02; 95% CI: 1.01-1.03), glucose (OR: 1.44; 95% CI: 1.04-1.99), and LDL cholesterol (OR: 1.38; 95% CI: 1.08-1.78) were independently associated with the presence of any CAC. With regard to the different degrees of CAC severity, in multivariable models, increasing age and male were significantly associated with all degrees of CAC severity. LDL cholesterol was associated with CAC <0-10 (OR: 1.60 [95% CI: 1.09-2.35]), SBP and glucose were associated with CAC <10-100 (OR: 1.02 [95% CI: 1.00-1.04] and OR: 1.56 [95% CI: 1.02-2.42], respectively), and SBP was associated with CAC >100 (OR: 1.02 [95% CI: 1.00-1.05] and OR: 2.04 [95% CI: 1.20-3.46], respectively). Sensitivity analysis using LDL <4.1 mmol vs ≥4.1 mmol showed similar associations with any CAC and CAC <0-10 (Table 3).

**Table 3:** Associations Between Cardiovascular Risk Factors and Presence of Any CAC (CAC >0), and Various CAC Severities

	Any CAC (CAC > 0)	CAC >0-10	CAC >10-100	CAC >100
Age	**1.12 (1.10-1.16)**	**1.09 (1.04-1.13)**	**1.12 (1.08-1.16)**	**1.20 (1.14-1.26)**
Male	**3.51 (2.32-5.53)**	**2.75 (1.37-5.53)**	**2.90 (1.64-5.13)**	**7.70 (3.25-18.2)**
Smoking	1.19 (0.59-2.44)	0.17 (0.02-1.38)	1.07 (0.42-2.75)	1.98 (0.69-5.69)
Body mass index	0.95 (0.88-1.02)	0.99 (0.89-1.11)	0.90 (0.82-1.00)	0.93 (0.82-1.07)
Systolic blood pressure	**1.02 (1.01-1.03)**	1.02 (1.00-1.04)	**1.02 (1.00-1.03)**	**1.02 (1.00-1.04)**
Glucose	**1.42 (1.04-1.95)**	0.98 (0.59-1.62)	**1.55 (1.01-2.37)**	**1.98 (1.19-3.27)**
LDL cholesterol	**1.39 (1.08-1.78)**	**1.61 (1.10-2.36)**	1.36 (0.98-1.90)	1.23 (0.78-1.95)
HDL cholesterol	0.96 (0.47-1.94)	0.87 (0.28-2.70)	0.67 (0.26-1.70)	1.61 (0.48-5.39)

Values are odds ratio (95% confidence interval). Values in **bold** indicate *P <* 0.05. Model adjusted for age, sex, smoking, sedentary behavior, body mass index, systolic blood pressure, glucose, LDL cholesterol, and HDL cholesterol.

Abbreviations as in Table 2.

**Figure 1:**
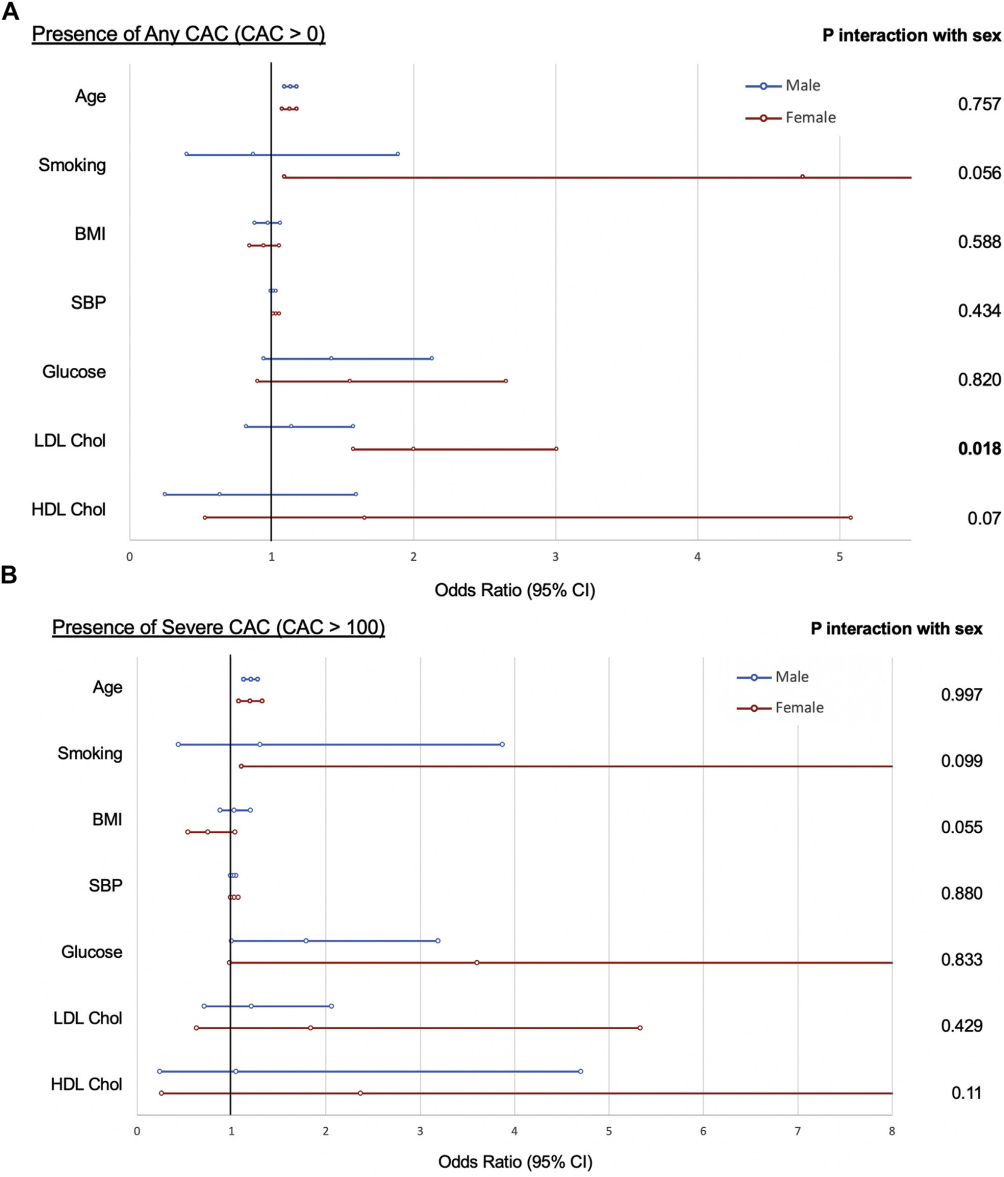
Sex-Stratified Associations Between Cardiovascular Risk Factors and Presence of Any CAC or Severe CAC **(A)** The associations between various cardiovascular risk factors and the presence of any CAC (CAC score >0), stratifying by sex. LDL cholesterol is shown to be significantly associated with any CAC in women, but not men. **(B)** The associations between various cardiovascular risk factors and the presence of severe CAC (CAC score >100), stratifying by sex. No differential associations are observed. BMI = body mass index; HDL = high-density lipoprotein; LDL = low-density lipoprotein; SBP = systolic blood pressure.

In men, only age was independently associated with any CAC (OR: 1.13; 95% CI: 1.09-1.18). In women, multivariable-adjustment models demonstrated that age, smoking, sedentary behavior, SBP, and LDL cholesterol were associated with the presence of any CAC. Interaction analysis revealed differential associations of LDL cholesterol with any CAC between sexes, with LDL associated with CAC more in women than men (adjusted *P*_interaction_ = 0.022) (Figure 1, Table 4).

**Table 4:** Gender-Stratified Associations Between Cardiovascular Risk Factors and Presence CAC

	Men		Women		Interactions	
	Univariate (95% CI)	Multivariate∗ (95% CI)	Univariate (95% CI)	Multivariate∗ (95% CI)	Univariate (95% CI)	Multivariate∗ (95% CI)
Presence of CAC (CAC > 0)						
Age	**1.14 (1.10-1.18)**	**1.13 (1.09-1.17)**	**1.15 (1.11-1.20)**	**1.12 (1.07-1.17)**	0.616	0.757
Smoking	0.91 (0.46-1.80)	0.87 (0.40-1.89)	1.54 (0.40-5.85)	**4.74 (1.09-20.7)**	0.489	0.056
BMI	0.97 (0.91-1.04)	0.97 (0.88-1.06)	0.96 (0.89-1.04)	0.94 (0.84-1.05)	0.875	0.588
SBP	**1.02(1.01-1.04)**	1.01 (0.99-1.03)	**1.04 (1.03-1.05)**	**1.03 (1.01-1.05)**	0.142	0.434
DBP	1.01 (0.99-1.04)	—	**1.04 (1.02-1.06)**	—	0.126	
Glucose	**1.55 (1.06-2.27)**	1.42 (0.94-2.13)	**1.95 (1.23-3.08)**	1.55 (0.90-2.65)	0.458	0.820
Total cholesterol	1.09 (0.86-1.38)	—	**2.29 (1.68-3.12)**	—	**0.000**	
LDL-cholesterol	1.11 (0.85-1.45)	1.14 (0.82-1.57)	**2.35 (1.65-3.34)**	**2.00 (1.44-3.00)**	**0.001**	**0.018**
HDL-cholesterol	0.90 (0.43-1.89)	0.63 (0.25-1.59)	**3.76 (1.64-8.62)**	1.59 (0.52-4.84)	**0.012**	0.07
Presence of severe CAC (CAC > 100)						
Age	**1.20 (1.14-1.28)**	**1.21 (1.13-1.28)**	**1.24 (1.13-1.36)**	**1.20 (1.08-1.33)**	0.637	0.997
Smoking	1.50 (064-3.51)	1.30 (0.44-3.87)	2.69 (0.32-23.0)	19.4 (1.11-340)†	0.620	0.099
BMI	1.00 (0.91-1.10)	1.03 (0.88-1.20)	0.82 (0.66-1.02)	0.75 (0.54-1.04)	0.102	0.055
SBP	**1.04 (1.02-1.07)**	1.02 (0.99-1.05)	**1.04 (1.02-1.07)**	1.03 (0.99-1.07)	0.867	0.880
DBP	1.03 (1.00-1.06)	—	1.03 (0.99-1.07)	—	0.951	
Glucose	**1.66 (1.06-2.59)**	**1.79 (1.00-3.19)**	**2.09 (1.07-4.09)**	3.60 (0.98-13.3)	0.572	0.833
Total Cholesterol	1.18 (0.85-1.63)	—	**2.44 (1.39-4.28)**	—	**0.028**	
LDL-cholesterol	1.14 (0.79-1.65)	1.21 (0.71-2.06)	**2.04 (1.10-3.79)**	1.84 (0.63-5.33)	0.113	0.429
HDL-cholesterol	1.18 (0.42-3.34)	1.05 (0.24-4.70)	**10.4 (2.06-52.9)**	2.37 (0.26-21.9)	**0.027**	0.11

BMI = body mass index; DBP = diastolic blood pressure; SBP = systolic blood pressure; other abbreviations as in Table 2.

∗ Model adjusted for age, gender, smoking, sedentary behavior, BMI, systolic BP, glucose, LDL cholesterol, and HDL cholesterol.

† Effect estimates out of proportion caused by limited sample size of female smokers.

Participants with higher CAC scores were likely to have higher 10-year CHD risk scores (Fisher’s exact *P* < 0.001). In particular, 16.7% of participants with CAC >100 had 10-year CHD risk scores of ≥5%, in comparison to just 1.5% of participants with CAC = 0 (*P <* 0.001) (Supplemental Table 1). After the incorporation of CAC scores, there was a significant increase in the mean ± SD 10-year CHD risk scores of our population from 1.78 ± 0.01% to 2.20 ± 0.01% (*P <* 0.001). Furthermore, we observed significant reclassification of individual risk, with 35 individuals (5.56%) reclassified from a “low” to an “elevated” risk of CHD (*P <* 0.001) (Supplemental Table 2).

## Discussion

In this cross-sectional study of 663 healthy individuals, the overall prevalence of preclinical coronary atherosclerosis (as measured by CAC) was 29.3%, with 9.0% having moderate-severe degrees of CAC. The prevalence of coronary atherosclerosis was higher in men than women, and increased with age, both overall and within each sex. We also observed sex-specific differences in the associations of LDL cholesterol with preclinical atherosclerosis. The CAC score has emerged as one of the most robust predictors of coronary events in an asymptomatic population, with strong predictive power demonstrated across both different ethnicities and sexes (10,14). These findings may contribute toward a better understanding of the development and progression of atherosclerosis within healthy individuals, and may enable improved sex and age-specific cardiovascular risk assessment and optimized intervention strategies to prevent progression into clinical CAD. Such data may especially be useful within Asian populations, wherein there still remains a relative paucity of population-based data on the distribution of preclinical atherosclerosis (26), despite Asia rapidly becoming an epicenter for CVD in the world (27, 28, 29).

Our reported prevalence of 29.3% is similar to the rates reported in the MESA study (33.7% in the nondiabetic healthy subset of the MESA cohort) (30), although it should be noted that the mean age of the MESA cohort (62 years) was higher than ours (49.4 years). Other large studies conducted on similarly aged populations, Lee et al. (31) (34%, mean age 53.8 years), VanWagner et al. (32) (27.1%, mean age 50.1 years), and Kim et al. (33) (31.1%, mean age 54 years) all also reported similar prevalence values to ours. We also observed a higher burden of preclinical atherosclerosis in men compared with women, and with the prevalence increasing with age in both sexes. Such trends have similarly been observed not just at the level of clinical CAD (8,34), but also at the preclinical level (in terms of CAC scores, or increased carotid intima-media thickness) in other populations (30,35,36). The prevalence of mod-severe CAC in women predominantly demonstrated an increase in the aged ≥60 years group, whereas in men this rise in prevalence was observed from age ≥40 years. It is well cited that significant CAD often occurs at a later age in women than men, with women tending to be 10 years older than men at time of presentation with CAD (4,5). We have shown that this relationship extends back to preclinical coronary atherosclerotic burden.

Age and sex were consistently independently associated with the presence of coronary atherosclerosis, both overall and across the various CAC severity subgroups. SBP, glucose, and LDL cholesterol were also found to be associated with coronary atherosclerosis. It should be noted that even within nondiabetic individuals, increased glucose levels still showed an association with increased risk. Smoking and BMI were not associated with CAC within our multivariable models, although this can potentially be explained by the low prevalence of smokers (7.69%) and obese individuals (5.3%) within our healthy population. Similarly, although HDL cholesterol was likewise not associated with CAC, recent evidence has suggested that HDL cholesterol levels may not necessarily be predictive of cardiovascular outcomes in all populations, with calls for a shift in focus away from absolute HDL concentrations toward more specific functions of HDL (37, 38, 39, 40).

Gender-specific differences in atherogenic risk factors were also observed in our study, with significant interactions with LDL cholesterol levels. LDL was more significantly associated with a positive CAC score in women compared with men in this study, although LDL levels are traditionally found to have a greater atherogenic risk effect in men than women (7). We posit 2 potential explanations for this. First, over one-half of our female participants were ≥50 years, which may possibly be a consequence of the loss of the protective effects of estrogen caused by menopause on the traditional CAD risk factors (6,7,41). Alternatively, there might have been a form of selection bias, wherein older men with higher risk lipid profiles may have already manifested clinical CAD events, and hence were not eligible for our healthy population.

It should be noted, however, that atherogenesis is a complex process, and sex-specific differences in atherogenic risk factors extend past differences in lipid profiles alone and may also include differences in systemic and vascular inflammatory profiles (8,42,43). Evidence also indicates that sex hormones (and the lack thereof postmenopause) have a role in modulating the immune response, resulting in different atherosclerotic phenotypes across sexes (6,44,45). However, literature specifically examining sex differences in the role of inflammatory mechanisms, mediators, and markers during the atherogenic process remain sparse (44). Regardless of these potential sex differences in proatherogenic inflammatory and lipid profiles, firm evidence exists for the role of lipid-lowering statins, which are also known to have anti-inflammatory effects, in the primary prevention of future cardiovascular events (46).

We also observed significant reclassifications in the cardiovascular risk of asymptomatic individuals upon incorporation of the CAC score. A total of 35 individuals (5.56%) within our population were re-classified from a “low” to an “elevated” risk of CHD. Such individuals might otherwise have fallen under the typical thresholds for cardiovascular disease risk factor monitoring. Multiple studies on the MESA cohort have previous demonstrated that the addition of CAC scores to prediction models significantly improved the classification of risk, and placed more individuals in higher-risk categories (21,47). The CAC score has potential utility in improving the risk stratification for CAD in asymptomatic healthy Asian populations, and this will be the work of further research.

### Study limitations

Limitations of our study include the cross-sectional nature of our study. The CAC score is a surrogate marker of subclinical atherosclerotic disease, and further research on actual clinical outcomes is required. As part of the SingHEART study, these patients are currently being followed up for long-term clinical events, and these clinical outcomes will subsequently be reported. There is also a potential for bias, as these participants were volunteers recruited from the general population; such individuals might have a higher regard for their health and potentially have more favorable health-related lifestyle choices. It should also be noted that the interpretation of CAC scores may be complicated by variations across sex and ethnic groups (10). We acknowledge that the lower prevalence of moderate-severe CAC (CAC >100) within women may not necessarily translate to a lower risk of CAD in such individuals, as age-sex specific percentiles, rather than the traditional absolute thresholds may be more important in translating CAC to cardiovascular risk (48,49).

## Conclusions

In this healthy asymptomatic population, preclinical coronary atherosclerosis was present in about 3 of 10 subjects. This prevalence increased with age, and was higher in men than women, with sex-specific differences in associated risk factors. The potential utility of the CAC score in CAD risk stratification was also highlighted. These results will better enable the optimization of age and sex-specific risk management strategies to prevent the development and progression of clinical CAD in healthy individuals.

Perspectives **COMPETENCY IN MEDICAL KNOWLEDGE:** Even within healthy asymptomatic Asian individuals, preclinical coronary atherosclerosis is present in about 3 of 10 subjects. The prevalence of preclinical coronary atherosclerosis in asymptomatic Asian individuals increases with age, is higher in men than women, and is subject to sex-specific differences in associated risk factors.

**COMPETENCY IN PATIENT CARE AND PROCEDURAL OUTCOMES:** The CAC score has the potential to improve the risk stratification for CAD in asymptomatic healthy Asian populations.

## Funding Support and Author Disclosures

The Lee Foundation provided grant support for the SingHeart study conducted at National Heart Centre Singapore. This work was also supported by core funding from SingHealth and Duke NUS through their institute of Precision Medicine (PRISM) and by a center grant awarded to National Heart Centre Singapore from the National Medical Research Council, Ministry of Health, Republic of Singapore (NMRC/CG/M006/2017_NHCS). Dr Yap has received honoraria fees (all modest) from Johnson and Johnson, Terumo and Amgen. Dr Yeo has received research funding from Medtronic, Boston Scientific, Amgen, AstraZeneca, and Shockwave Medical (all significant, via institution); has received consulting or honoraria fees (all modest) from Medtronic, Boston Scientific, Abbott Vascular, Amgen, Bayer, and Novartis; and has received speaker or proctor fees from Abbott Vascular, Boston Scientific, Medtronic, Philips, Shockwave Medical, Alvimedica, Menarini, AstraZeneca, Amgen, and Bayer. All other authors have reported that they have no relationships relevant to the contents of this paper to disclose.
