# Supplementary material for: Impact of Age and Sex on Subclinical Coronary Atherosclerosis in a Healthy Asian Population
Source: JACC Asia. 2021 Jun 15;1(1):93–102. doi: 10.1016/j.jacasi.2021.05.002 (PMC9627875; doi:10.1016/j.jacasi.2021.05.002)
Supplement: Supplemental Tables 1 and 2 [file mmc1.docx]

**Supplemental Table 1. Prevalence of CAC severities by MESA 10-year CHD Risk classification**

|  | **MESA 10-year CHD Risk scores (without CAC incorporation)** | | | |  |
| --- | --- | --- | --- | --- | --- |
| **CAC Scores** | **<2.5%** | **2.5 - 4.99%** | **5 – 7.44%** | **≥7.5%** | **P value** |
| **0, n(%)** | 409 (87.2) | 53 (11.3) | 6 (1.28) | 1 (0.21) | <0.001 |
| **>0-10, n(%)** | 30 (62.8) | 15 (31.9) | 2 (4.26) | 0 (0.0) |  |
| **>10-100, n(%)** | 48 (55.2) | 34 (39.1) | 5 (5.75) | 0 (0.0) |  |
| **>100, n(%)** | 17 (28.3) | 33 (55.0) | 6 (10.0) | 4 (6.67) |  |

**Supplemental Table 2. MESA 10-year CHD Risk classification, before and after incorporation of CAC scores**

|  | | **MESA risk with incorporation of CAC** | |
| --- | --- | --- | --- |
|  |  | 10-Year CHD risk <7.5% | 10 Year-CHD risk ≥7.5% |
| **MESA Risk without incorporation of CAC** | 10-Year CHD risk <7.5% | 623 (94.0%) | 35 (5.56%) |
|  | 10-Year CHD risk ≥7.5% | 1 (0.12%) | 4 (0.6%) |
